# Interleukin-9 promotes cell survival and drug resistance in diffuse large B-cell lymphoma

**DOI:** 10.1186/s13046-016-0374-3

**Published:** 2016-07-01

**Authors:** Xiao Lv, Lili Feng, Xueling Ge, Kang Lu, Xin Wang

**Affiliations:** Department of Hematology, Shandong Provincial Hospital Affiliated to Shandong University, Jinan, Shandong 250012 China

**Keywords:** Interleukin-9, Diffuse large B-cell lymphoma, Drug resistance, P21CIP1

## Abstract

**Background:**

Interleukin-9 (IL-9) was discovered as a helper T cell growth factor. It has long been recognized as an important regulator in allergic inflammation. Recent years it was discovered to induce cell growth and differentiation of multiple transformed cells. However, its oncogenic activities in B-cell lymphomas have not been reported in detail.

**Methods:**

Serum levels of IL-9 in DLBCL patients were quantified by ELISA, and its clinical significance was analysed. The expression of IL-9 receptor (IL-9R) was investigated in lymphoma cell lines by RT-PCR and western blot, respectively. In DLBCL cell lines LY1 and LY8, IL-9R genes were knocked down by RNA interference and stable transfected cells were selected with puromycin. Normal and final siIL-9R (and siControl) LY1 and LY8 cells were treated with IL-9 alone and in synergy with chemotherapeutic drugs. Cell proliferation and apoptosis were assessed by Brdu incorporation and flow cytometric analysis. The mRNA of apoptosis regulation genes were measured with real-time PCR.

**Results:**

Elevated serum levels of IL-9 were detected in DLBCL patients (24/30) compared to healthy controls (0/15). Positive expression of IL-9 (defined as a serum level ≥1 pg/ml) was correlated with lower serum albumin levels and high international prognostic index (IPI) scores. IL-9R was expressed in both mRNA and protein levels in the five lymphoma cell lines, including LY1, LY8, MINO, SP53 and Jurkat. In vitro studies showed that IL-9 directly induced proliferation and inhibited apoptosis in LY1 and LY8 cells. It protects LY1 and LY8 cells from prednisolone induced apoptosis, and promotes their proliferation that were inhibited by rituximab, vincristine and prednisolone. Its molecular mechanism may be concerned with upregulating expression of p21CIP1 gene. Knock-down of IL-9R gene could reverse the effects of IL-9 on LY1 and LY8 cells.

**Conclusions:**

IL-9 is associated with clinical features of DLBCL patients. It promotes survival of DLBCL cells and reduces the sensitivities of tumor cells to chemotherapeutic drugs via upregulation of p21CIP1 genes.

**Electronic supplementary material:**

The online version of this article (doi:10.1186/s13046-016-0374-3) contains supplementary material, which is available to authorized users.

## Background

Diffuse large-B-cell lymphoma (DLBCL) is the most common form of non-Hodgkin’s lymphoma (NHL) in adults. It accounts for about 30 % of total NHL cases with an annual incidence of 7–8 cases per 100, 000 of the population [1, 2]. Standard chemotherapy with cyclophosphamide, doxorubicin, vincristine and prednisone (CHOP) has helped over 40 % of DLBCL patients achieve long-term survival. The addition of rituximab to the CHOP regimen increased the 5-year overall survival to nearly 60 %. Although tremendous progress has been made in the outcomes of DLBCL, approximately one third of patients died from drug resistance or from relapse [3]. The unclear molecular etiology of the disease limits further development of DLBCL therapy.

The biologic processes that lead to lymphomagenesis are complex and seem to be different among lymphoma histotypes. It has been widely acknowledged that immune system dysfunction plays a crucial role in the pathogenesis of lymphoma [4, 5]. Abnormal production of cytokines, which serve crucial regulatory roles in host immunity, accompany the onset of certain lymphomas [6]. The cytokine IL-9 was initially described as a growth factor secreted by activated Th2 cells. It acts through a γC-family receptor on target cells and has diverse functions in immune and inflammatory responses [7–9]. Recently, the determination of the growth-promoting and anti-apoptotic activities of IL-9 in multiple transformed cell lines suggests a potential role of IL-9 in tumorigenesis [10]. Dysregulated expression of IL-9 was detected in select sub-populations of Hodgkin’s Disease (HD) and nasal natural killer (NK)/T-cell lymphoma patients [11, 12]. Experimental analysis of tissue sections also demonstrated a unique association between IL-9 expression and the development of HD and anaplastic large cell lymphoma (ALCL) [13]. However, the oncogenic activities of IL-9 in lymphomas derived from B-cell lineages have not been reported in detail.

Our previous study demonstrated that there was an elevated serological level of IL-9 in some B-cell NHL patients (including several DLBCL cases). Neutralizing IL-9 with certain specific antibodies inhibited tumor growth in murine models of B-cell lymphoma [14]. Consequently, we formally considered the possibility that IL-9 might contribute to the development of DLBCL. In an attempt to validate this hypothesis, we examined the expression of IL-9 in the sera of DLBCL patients, and demonstrated the effect of IL-9 on the biological behavior of DLBCL cell-lines in vitro.

For the first time, we provide convincing evidence of the participation of IL-9 in DLBCL and offer the notion that specific silencing of the IL-9R gene may serve a potentially therapeutic approach in the clinical management of DLBCL.

## Methods

### Patients

Blood samples from 30 DLBCL patients were taken at diagnosis while sera from 15 healthy volunteers served as normal controls. Clinical information of the enrolled DLBCL patients, including sex, age, International Prognostic Index (IPI) score, serum levels of lactate dehydrogenase (LDH), albumin and β2 microglobulin, were also collected. All DLBCL cases were diagnosed between January 2010 and May 2014 according to the WHO criteria [15].

### ELISA for IL-9

Serum samples from 30 DLBCL patients and 15 healthy volunteers were collected and frozen at −80 °C. Serological levels of IL-9 were quantified using a human ELISA kit (eBioscience) according to the manufacturer’s instructions.

### Cell culture

The human DLBCL cell-lines LY1 and LY8 [16] were cultured in IMDM supplemented with 10 % fetal bovine serum. The two human mantle cell lymphoma (MCL) cell-lines Mino and SP53 (a kind gift from Dr. Michael Wang, Department of Lymphoma and Myeloma, The University of Texas, MD Anderson Cancer Center), the human acute T cell Leukemia cell-line Jurkat, and the human myeloma cell-line RPMI8226 (obtained from the China Center for Type Culture Collection) were maintained in RPMI-1640 medium supplemented with 10 % FBS. Stable transfected cells were grown in IMDM supplemented with puromycin (2 g/mL) and 10 % FBS. All cell-lines were incubated at 37 °C in an atmosphere containing 5 % CO2.

### Western blot analysis

The expression of IL-9R in five lymphoma cell lines was determined by Western blot analysis. Total protein was extracted by RIPA and 1 % PMSF. The protein concentrations were determined by the BCA assay. Cell lysates were then resolved by electrophoresis on a 10 % SDS-polyacrylamide gel and electro-transferred onto nitrocellulose membranes. After the membranes were blocked with 5 % skim milk non-fat proteins in Tris-saline buffer that was supplemented with 0.1 % Tween-20, they were subsequently probed with primary antibodies (i.e., rabbit anti-IL-9Rα polyclonal antibody, 1:100, Abcam, and mouse anti-β-actin monoclonal antibody, 1:10,000; Abcam) at 4 °C overnight. After washing with TBST, secondary antibody that was conjugated with horseradish peroxidase (1:8000), was added to the membranes. Subsequently, proteins were detected by an enhanced chemiluminescence detection kit (Millipore).

### Reverse transcription PCR and real-time quantitative PCR

Total RNA was extracted from the cell lines using Trizol reagent (Invitrogen) according to the manufacturer’s instructions. Reverse transcription was performed using the PrimeScript RT (reverse transcription) reagent Kit (TaKaRa). The expression of IL-9R mRNA in lymphoma cell lines was detected by RT-PCR with the cycling parameters defined as follows: an initial cycling for 5 min at 94 °C, followed by 40 cycles of 15 s at 95 °C, 30 s at 59 °C and 55 s at 72 °C. PCR products were confirmed as a single product of the desired size on agarose gels and visualized by ethidium bromide (EtBr) staining.

In addition, the changes in the mRNA levels for MYC, BAX, p21CIP1, PIM1, BCL2L1 and MCL1 after IL-9 treatment were measured by using real-time quantitative PCR with a SYBR Premix Ex Taq Kit (TaKaRa). Samples were run in triplicate under standard conditions (95 °C for 30 s followed by 40 cycles at 95 °C for 5 s and 65 °C for 15 s) in a Roche 480 instrument. The relative expression of each gene was quantified by using β-actin expression as an endogenous control. Dissociation curves were performed after each experiment to confirm the specificity of product amplification. The data collated for mRNA relative fold increase was analyzed using untreated cells as a reference control. Specific primers for the PCR assay were obtained from Biosune, and the primer sequences are shown in Table 1.

**Table 1:** Primer sequences of apoptosis related genes

Gene name	Sequence
IL-9R	5′-CGTGCCCTCTCCAGCGATGTTCT-3′
	5′-GACGCGCTGGGCCACAAGTG -3′
MYC	5′-GGCTCCTGGCAAAAGGTCA-3′
	5′-AGTTGTGCTGATGTGTGGAGA-3′
BAX	5′-CCCGAGAGGTCTTTTTCCGAG-3′
	5′-CCAGCCCATGATGGTTCTGAT-3′
p21CIP1	5′-CGATGGAACTTCGACTTTGTCA-3′
	5′-GCACAAGGGTACAAGACAGTG-3′
PIM1	5′-GAGAAGGACCGGATTTCCGAC-3′
	5′-CAGTCCAGGAGCCTAATGACG-3′
BCL2L1	5′-TCAGAGCTTTGAGCAGGTAG-3′
	5′-AAGGCTCTAGGTGGTCATTC-3′
MCL1	5′-CGCAACCACGAGACGG -3′
	5′-TCACATCGTCTTCGTTTTTGAT -3′
β-actin	5′-TGACGTGGACATCCGCAAAG -3′
	5′-CTGGAAGGTGGACAGCGAGG-3′

### Knockdown of IL-9R by lentivirus-mediated RNA interference

Knockdown of the IL-9R gene was carried out by lentivirus-mediated RNA interference. Oligonucleotides coding for the short hairpin RNA (shRNA) targeting human IL-9R (with a target sequence of 5′- GCTCGTGCCATCTGACAATTT-3′) were annealed and inserted into the pLK-GFP-puro vector (Shanghai Telebio company, China). The empty pLK-GFP-puro vector was employed as a negative control. The pLK-shRNA and control vector were then transfected into HEK293 cells, together with second generation packaging plasmids (i.e., pRsv-REV, pMDlg-pRRE, pMD2G). Virus supernatant was collected 72 h after transfection. LY1 and LY8 cells were infected with the IL-9R-RNAi or negative control lentivirus at a multiplicity of infection (MOI) of 80. The stably infected cells were selected with puromycin (5 g/mL) for 2 weeks to obtain final siIL-9R and siControl LY1 and LY8 cells. The efficiency of IL-9R knockdown was assessed by both real-time PCR and Western blot analysis (Additional file 1: Figure S1).

### Cell treatment

To test the direct influence of IL-9 on DLBCL cells, LY1 and LY8 cells were cultured dose-dependently with IL-9 (0, 20, 40, 60 and 80 ng/mL) for 72 h, following which, cellular apoptosis was analyzed by flow cytometry. Moreover, cell proliferation was also tested after LY1 and LY8 cells were treated with IL-9 (80 ng/mL) for 72 h.

To determine the effect of IL-9 on the responses of DLBCL cells to chemotherapeutic drugs, stably transfected (i.e., siIL-9R and siControl) LY1 and LY8 cells were exposed to prednisone (100 ug/ml), rituximab (10 ug/ml) and vincristine (0.8 ng/ml) either in the presence or absence of IL-9 (80 ng/mL) for 72 h. After these interventions, cell proliferation and apoptosis were evaluated by BrdU incorporation and FACS analysis, respectively.

### Flow cytometric analysis of cell apoptosis

Cell death was measured by flow cytometry using an annexin V- FITC and propidium iodide (PI) apoptosis detection kit (KeyGEN BioTECH) according to the manufacturer’s instructions. Briefly, 10^5^ to 5 × 10^5^ cells were incubated with annexin V-FITC and PI for 10 min at room temperature in the dark. Cells were then immediately analyzed using a FACScan flow cytometer (BD Biosciences). Viable cells were not stained with annexin V-FITC or PI, while by contrast, necrotic cells were stained annexin V-FITC and PI positive, whereas apoptotic cells were stained annexin V-FITC-positive and PI-negative [17, 18].

### Cell proliferation assays

Cell proliferation was tested by BrdU incorporation. Cells were grown at a density of 1 × 105/ml in 96-well plates. After 72 h of treatment, cell proliferation was measured using the BrdU Cell Proliferation ELISA (Roche) according to the manufacturer’s protocols. The absorbance of each well was determined at a wavelength of 450 nm using an automated microplate reader.

### Statistical analysis

The numerical data were presented as mean ± SD. All statistical analyses were performed by using the statistics software program SPSS version 16.0 for Windows. Independent-sample T test was used to analyze numerical variables. Non-parametric data and comparative analysis between both groups of patients with different IL-9 expression were evaluated by Chi-square analysis and Fisher’s exact test. Statistically significant differences were defined at an alpha value of *P* < 0.05.

## Results

### Upregulated serological IL-9 of DLBCL patients and its correlation with clinical characteristics

**Fig. 1:**
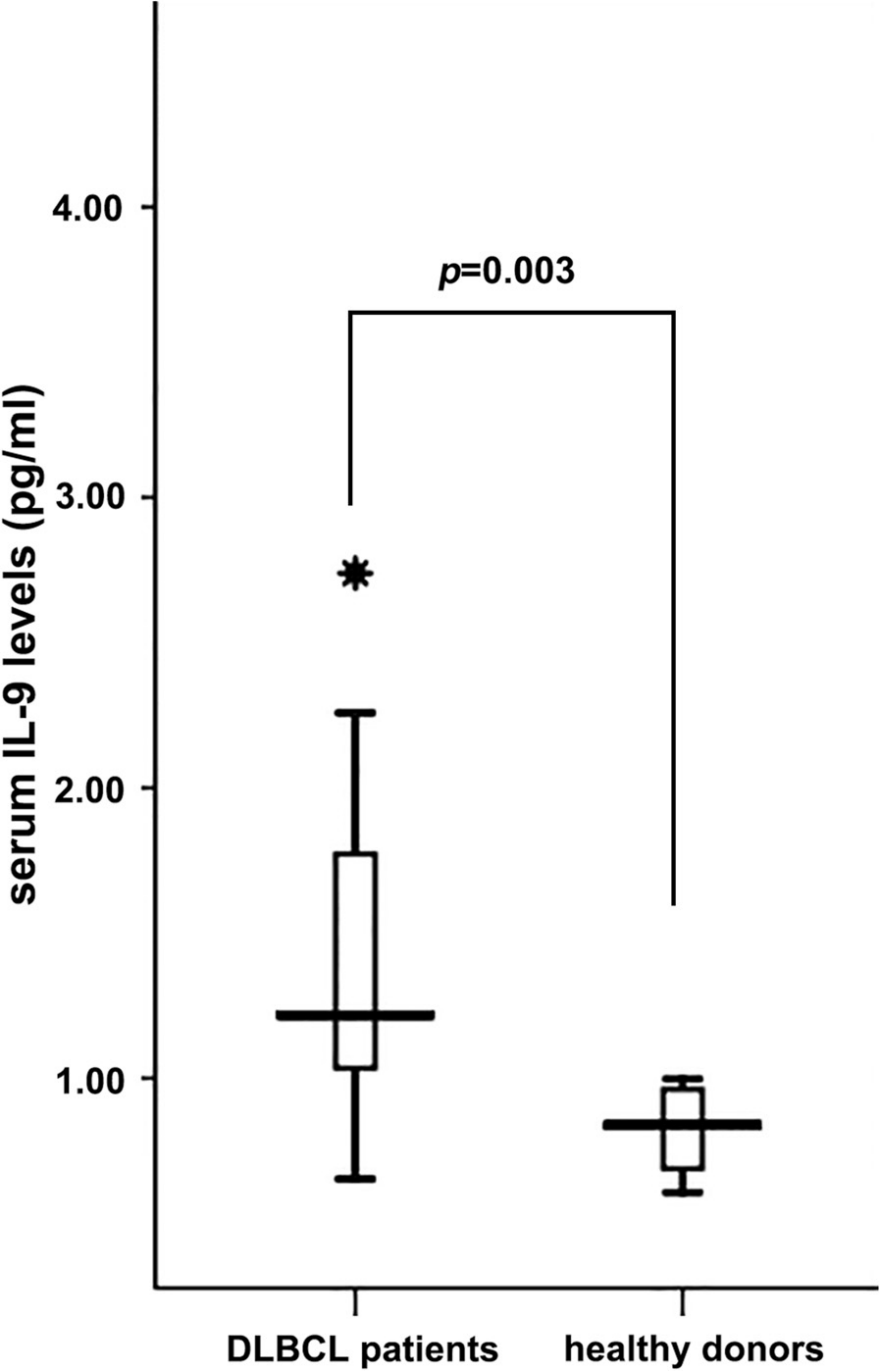
Serum levels of IL-9 were higher in DLBCL patients (1.43 ± 0.64 pg/ml) than healthy controls (0.82 ± 0.15 pg/mL). If IL-9 concentration ≥1 pg/ml was defined as positive expression, Fisher’s exact test revealed significant differences between the two groups (*p* = 0.003)

To investigate the possible involvement of IL-9 in DLBCL, we compared serum levels of IL-9 in patients with DLBCL and healthy controls (Fig. 1). If the serum IL-9 level ≥1 pg/ml was defined as positive expression, IL-9 was detected in 24 of 30 sera from DLBCL patients (mean concentration 1.43 ± 0.64 pg/ml) while no serum samples from healthy volunteers contained marginally detectable levels of IL-9 (0.82 ± 0.15 pg/ml). Fisher’s exact test revealed significant differences between both groups (*p* = 0.003).

In addition, the clinical characteristics between DLBCL patients with different IL-9 expressions were also compared. As shown in Table 2, there were no differences in age (*P* = 0.848) and sex (*P* = 0.678) between groups. The association between positive IL-9 expression (concentration ≥1 pg/ml) and serum levels of LDH (506.3 vs 279.2 u/L, *P* = 0.945) and β2 microglobulin (2.35 vs 1.8 ug/ml, *P* = 0.202) was also not statistically significant. Nevertheless, patients with positive IL-9 expression showed a lower serum albumin level (35.85 ± 3.65 vs 41.8 ± 2.12 g/L, *P* = 0.009). The international prognostic index (IPI) score, which is the most important prognostic indicator, was higher in IL-9 positive cases compared with IL-9 negative cases (*P* < 0.001).

**Table 2:** Correlation between IL-9 expression and clinical characteristics

Clinicopathological characteristics	IL-9 expression in sera		*P* value
	Negative (%)	Positive (%)	
Age (y)	57	55	0.776^a^
Sex			0.678^b^
Male	4 (50)	14 (63.6)	
Female	4 (50)	8 (36.4)	
Serum LDH level	279.2	506.3	0.945^a^
Serum β2 microglobulin level	1.8	2.35	0.202^a^
Serum albumin level	41.8	35.8	0.009^a^
IPI score			
0	6 (75)	0	<0.001^b^
1	0	0	
2	0	10 (62.5)	
3	2 (25)	2 (12.5)	
4	0	4 (25)	

^a^: Student’s t test t-test. ^b^ : Fisher’s exact test

### IL-9R expressed in lymphoma cell lines

**Fig. 2:**
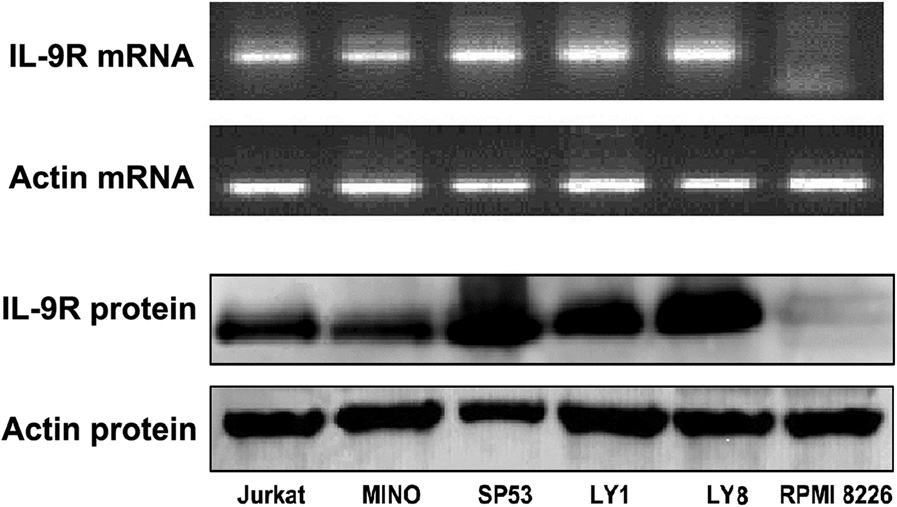
The expressions of IL-9R in lymphoma cell lines were tested by RT-PCR and western blot. All the five lymphoma cell lines (LY1, LY8, MINO, SP53 and Jurkat) were detected to express IL-9R in both mRNA and protein levels. Myeloma cell line RPMI8226 served as negative control

In our previous study, immunohistochemical analysis showed that the IL-9R protein was located on the membrane of tumor cells within DLBCL tissues. Overexpression of IL-9R protein was correlated with serum lactic dehydrogenase (LDH) levels, clinical stage and a high Ki-67 labeling index in DLBCL patients [19]. Concordant with in vivo observations, the expression of IL-9R in lymphoma cell-lines was confirmed by RT-PCR and western blot analysis, respectively. IL-9R expression was demonstrated for both mRNA and protein levels within the five lymphoma cell-lines, including LY1, LY8, MINO, SP53 and Jurkat (Fig. 2). The myeloma cell-line RPMI8226 served as a negative control.

### IL-9 directly induced proliferation and inhibited apoptosis in DLBCL cells

**Fig. 3:**
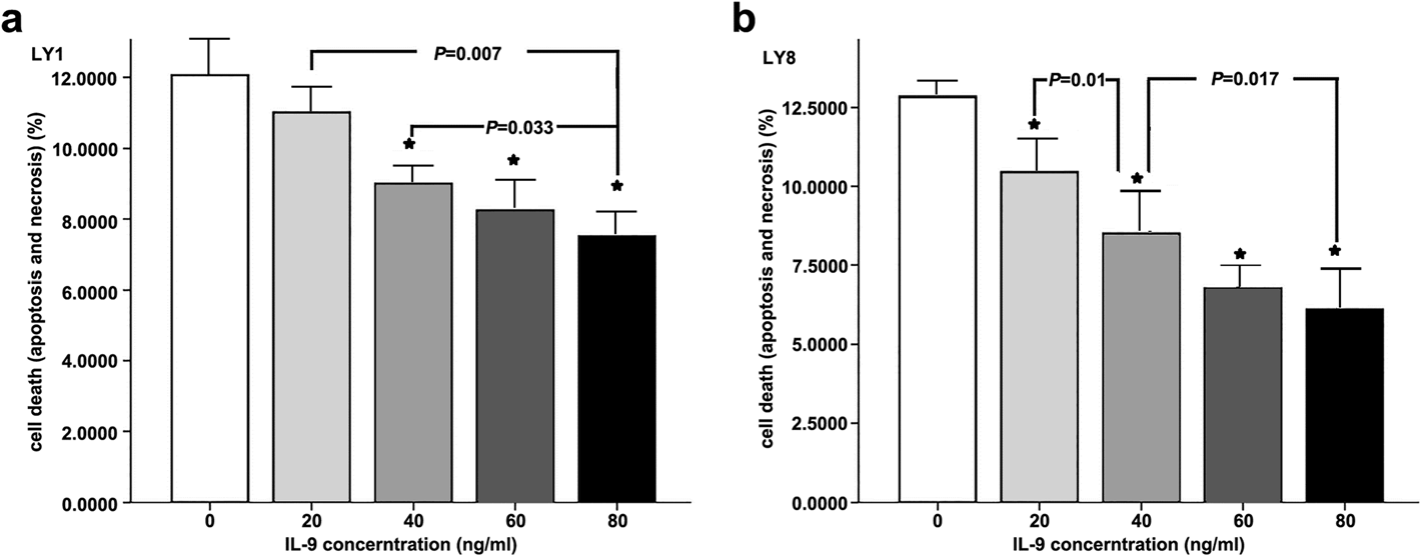
**a** LY1 cells. **b** LY8 cells. *The cell apoptosis at this IL-9 concentration was statistically significant compared to untreated cells (IL-9 concentration is 0). Cell apoptosis between groups at different IL-9 concentrations had statistical significance. This means that the apoptosis of LY1 and LY8 cells was dose-dependently reduced upon exposure to IL-9. IL-9 at a concentration of 80 ng/mL decreased cell apoptosis to approximately 70 % and 50 % of the baseline levels in LY1 and LY8 cells, respectively

To determine whether IL-9 activated intracellular signals, we treated LY1 and LY8 cells with IL-9 at different concentrations. As shown in Fig. 3, the apoptosis of LY1 and LY8 cells was dose-dependently reduced upon exposure to IL-9. At a concentration of 80 ng/mL, IL-9 decreased cellular apoptosis to approximately 70 and 50 % of the baseline levels in LY1 and LY8 cells, respectively.

**Fig. 4:**
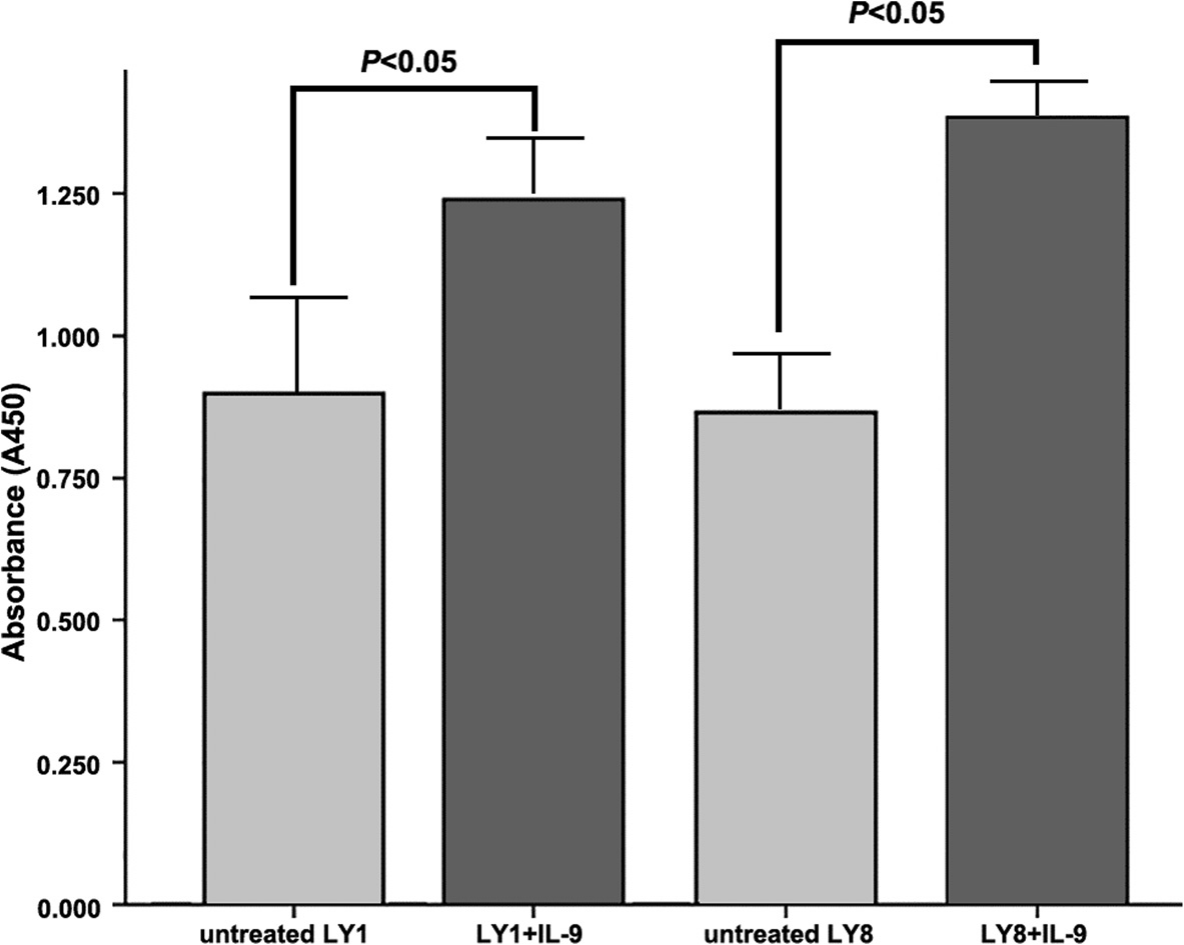
The proliferative activities were assessed by brdu incorporation. Direct IL-9 treatment on both LY1 and LY8 cells displayed statistically enhancement at absorbance of 450 nm

Besides, the proliferative responses of LY1 and LY8 cells in response to IL-9 were also assessed by measuring BrdU incorporation after 3 days of culture. Direct IL-9 treatment (80 ng/ml) of DLBCL cells displayed a marked increase in proliferation (Fig. 4). LY1 and LY8 cells showed an approximate 20 % increase in BrdU incorporation.

### IL-9 protects DLBCL cells from prednisolone induced apoptosis

**Fig. 5:**
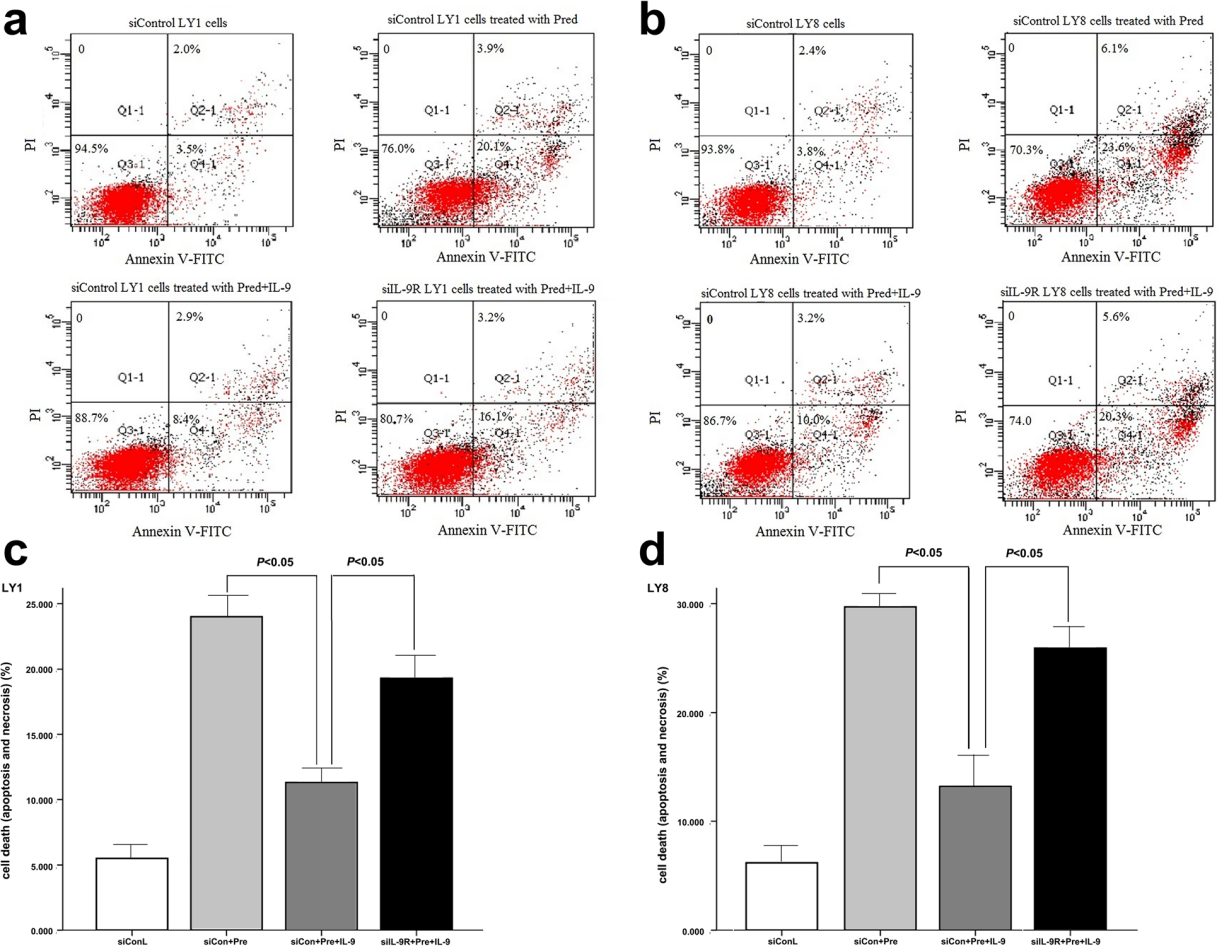
**a.c** LY1 cells. **b.d** LY8 cells. In both sicontrol LY1 and LY8 cells, IL-9 reduced apoptosis induced by prednisone. If IL-9R gene was knockdown by RNA interference, this protective effect was weakened

Stably transfected (siIL-9R and siControl) LY1 and LY8 cells were incubated with prednisolone (100ug/ml) for 72 h either in the presence or absence of IL-9 (80 ng/mL). Cell viability was measured by FACS after double-staining with annexinV and PI. As indicated in Fig. 5, the exposure to prednisone induced a significant cellular apoptosis in both siControl LY1 and LY8 cells; moreover, this process was almost completely inhibited by IL-9. However, in siIL-9R cells, whose IL-9R gene was silenced by RNA interference, the anti-apoptotic activity of IL-9 was not obvious.

### IL-9 promotes proliferation that was inhibited by chemotherapeutic drugs

**Fig. 6:**
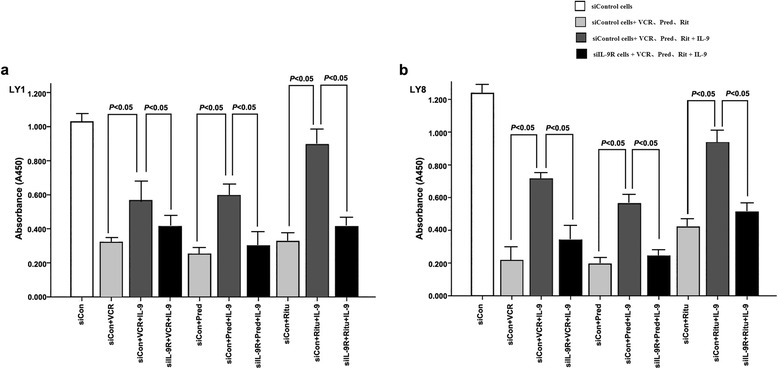
**a** LY1 cells. **b** LY8 cells. In siControl cells, the reduced cell proliferations induced by vincristine, prednisone and rituximab could be reversed by IL-9. The pro-proliferative effect was not obvious in siIL-9R cells

Stable transfected siControl LY1 and LY8 cells were exposed to prednisone (100ug/ml), rituximab (10ug/ml) and vincristine (0.8 ng/ml) for 72 h and then cell proliferation was evaluated. As shown in Fig. 6, all three drugs obviously inhibited cell proliferation. When we treated cells with IL-9 synergistically, the killing effect by chemotherapeutic drugs was weakened. However, in siIL-9R cells, the drug resistance that was induced by IL-9 was alleviated.

### IL-9 augments p21CIP1 expression in DLCBL cells

**Fig. 7:**
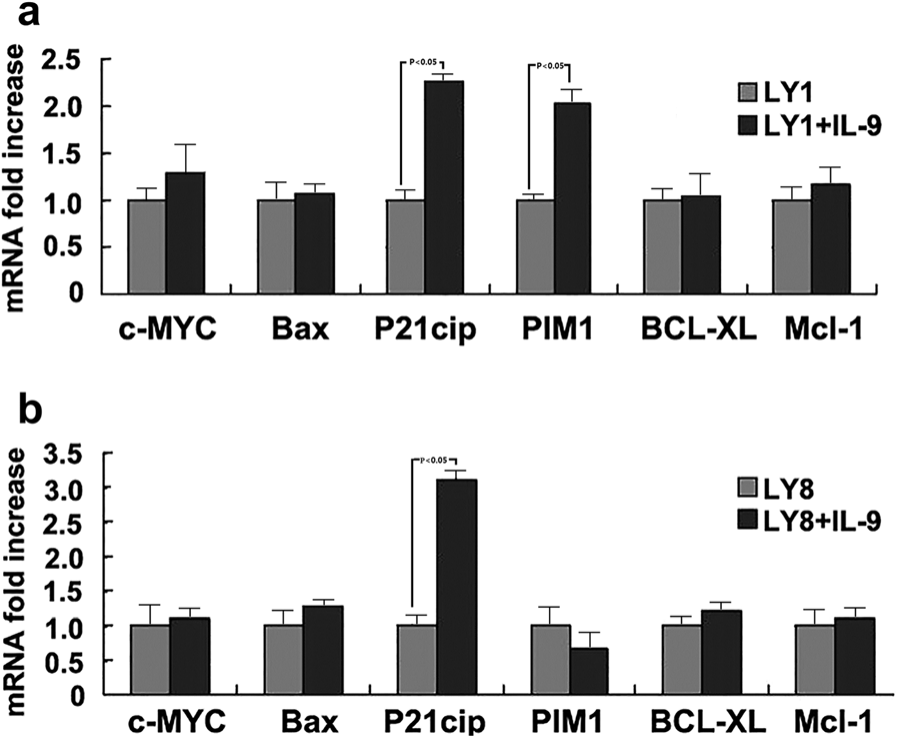
**a** LY1 cells. **b** LY8 cells. IL-9 influences the expression changes of apoptotic genes in DLBCL cells. The mRNA expression of p21CIP1 was upregulated by IL-9 to 2 folds in LY1 cells and more than 3 folds in LY8 cells. There were no significant changes of mRNA expression in anti-apoptosis genes including MYC, BAX, BCL2L1 and MCL1. The expression changes of PIM1 genes were different in LY1 and LY8 cells. It amplified to 2 folds in LY1 cells and remained unchanged in LY8 cells

To gain further insights into the molecular mechanism by which IL-9 promoted survival of DLBCL cells, we treated LY1 and LY8 cells with IL-9 (80 ng/ml) for 72 h and then we investigated the differential changes in expression of six JAK-STAT downstream regulatory genes, including MYC, BAX [20], p21CIP1, PIM1 [21, 22], BCL2L1 and MCL1 [23]. We analyzed data of mRNA fold increases using untreated cells as a reference control. As shown in Fig. 7, the mRNA expression of p21CIP1 was upregulated by two-fold in LY1 cells and by more than three-fold in LY8 cells. The expression changes of PIM1 genes were different in LY1 and LY8 cells. It amplified to 2 folds in LY1 cells and remained unchanged in LY8 cells. There were no significant changes in mRNA expression of anti-apoptosis genes including MYC, BAX, BCL2L1 and MCL1.

## Discussion

The biologic processes that lead to lymphomagenesis are complex and seem to be different among lymphoma histotypes. Numerous cytokines are believed to take part in this process [24, 25].

The dysregulated expression of IL-9 has been detected in biopsies or serum specimens of patients with some malignant lymphomas, such as HD, ALCL and NKT-cell lymphoma, which provides clinical evidence for its possible involvement in lymphomagenesis [13]. However, the pathogenic role of IL-9 in lymphomas derived from B-cell lineages has not been previously reported. In our previous study, IL-9 was demonstrated to take part in the pathogenesis of B-cell NHL by augmenting the extent of immunosuppression that is mediated by Treg cells and mast cells [14]. Based upon these results, we attempt to demonstrate that IL-9 plays a direct role in the survival of DLBCL cells.

Our studies indicate that serum levels of IL-9 are elevated in patients with DLBCL. The positive sera IL-9 expression is associated with some clinical parameters, including high IPI scores and low serum levels of albumin. High IPI means worse disease prognosis and low serum levels of albumin is usually connected with more severe disease consumption. These results imply that high serumal IL-9 levels may be correlated with more tumor burden.

In this study, we don’t analyse the exact source of IL-9. In fact, some studies have confirmed that IL-9 could act in an autocrine manner in ALCL and NKT-cell lymphomas [13]. Meanwhile, many researchers believe that certain immune cells, including Tregs, mast cells, Th7 and Th17 cells, could produce IL-9 [26]. Here, we think that the elevated IL-9 levels might be secreted by DLBCL cells themselves, and might also be produced by the non-malignant infiltrating cells within tumor tissues. Nevertheless, whatever source of IL-9 in the serum of patients, the correlation between augmented IL-9 levels and the more severe disease state provides direct clinical evidence for the contribution of IL-9 to the pathogenesis of DLBCL.

Since there was an upregulation of IL-9 in the sera of patients with DLBCL, we investigated the expression of IL-9R in DLBCL tissues and cell-lines to explore the actions of IL-9 on DLBCL cells. The IL-9R is comprised of a cytokine-specific α-chain and a common γ-chain, which is shared with IL-2, IL-4, IL-7, IL-15 and IL-21. The effects of IL-9 on target cells were mainly dependent on its high-affinity binding with the IL-9R. In our previous study, nearly 70 % of DLBCL cases examined stained positive for IL-9R by immunohistochemistry. Malignant DLBCL cells in tissue sections were confirmed by pathologists, and IL-9R was located on the surface of these tumor cells [19]. As shown by in vitro studied herein, the expression of IL-9R was detected at both the mRNA and protein levels within the five lymphoma cell-lines, including the DLBCL cell-lines LY1 and LY8, mantle cell lymphoma (MCL) cell-lines Mino and SP53, as well as the human acute T cell leukemia cell-line Jurkat. This may imply that the tumorigenic effect of IL-9 also exists in other types of lymphoma.

To examine the direct influence of IL-9 on DLBCL cell-lines, we treated LY1 and LY8 cells with exogenous rhIL-9 in vitro. The result indicates that there is a concentration-dependent decrease in cell apoptosis on both LY1 and LY8 cells. At the same time, a pro-proliferative activity of IL-9 was also observed after the intervention. These observations are consistent with clinical findings and provide direct evidence that IL-9 can promote the survival of DLBCL cells.

Since IL-9 plays a direct anti-apoptotic and pro-proliferative effect on DLBCL cells, we have considered the possibility that IL-9 might dampen the sensitivities of DLBCL cells to chemotherapeutic drugs. To examine the validity of this hypothesis, siControl LY1 and LY8 cells were exposed to prednisone, vincristine and rituximab in synergy with IL-9. The cytotoxic effect of vincristine on lymphoma cells is usually performed through inhibition of cell proliferation [27] and similar actions are also possessed by rituximab [28] and prednisone. Our studies demonstrated that the inhibited cell proliferation by these chemotherapeutic drugs was reversed by IL-9. Analysis of cell apoptosis reveals that IL-9 protects DLBCL cells from prednisone-induced apoptosis. These findings indicate that the lymphomagenic activity of IL-9 is still valid in the presence of chemotherapeutic drugs. On account that the effects of IL-9 on target cells are dependent on the high-affinity binding of IL-9 with its receptor, we attempted to knockdown the IL-9R gene by RNA interference. The results display that silencing of the IL-9R gene alleviates the drug resistance that is induced by IL-9, which provides a potential therapeutic target in DLBCL.

Our previous experiments have demonstrated that IL-9 promoted the survival of DLBCL cells. The signal transduction mediated by IL-9 and its receptor is mainly dependent on the JAK/STAT pathway [29, 30]. To gain further insight into the molecular mechanism of IL-9, we measured expression of its downstream genes. Real time RT-PCR revealed augmented levels of p21CIP1 genes in both LY1 and LY8 cells due to exposure to IL-9.

P21CIP1 is a cell-cycle regulatory protein that interacts with the cyclin-dependent kinases (CDK) CDK2 and CDK4 [31]. Moreover, P21CIP1 promotes the assembly of active cyclin D1/CDK complexes and stimulates cell cycle progression [32]. Enhanced expression of p21CIP1 provides a potential explanation for the pro-proliferative and anti-apoptotic activities of IL-9.

## Conclusions

In conclusion, our findings showed that serum levels of IL-9 were elevated in DLBCL patients and positive expression of IL-9 was correlated with adverse prognosis indicators. It directly effected proliferation and apoptosis of DLBCL cells by enhancing the expression of p21CIP1 genes and promoted tumor cells to display resistance to chemotherapeutic drugs. Knock-down of the IL-9R gene by RNA interference reversed the lymphomagenic activities of IL-9 on DLBCL cells. These observations contribute to our understanding of the cause and resistance mechanisms of DLBCL, and provide a potential targeted therapeutic approach for DLBCL.

## Abbreviations

ALCL, anaplastic large cell lymphoma; BrdU, 5-bromo-2′-deoxyuridine; CDK, cyclin-dependent kinases; DLBCL, diffuse large-B-cell lymphoma; IPI, international prognostic index; JAK-STAT, Janus kinase - signal transducer and activator of transcription; NHL, non-Hodgkin’s lymphoma

## Additional file

Additional file 1: Figure S1. IL-9R gene was knockout using lentivirus-mediated RNA interference. The efficiency of IL-9R knockdown was assessed by western blot analysis. The expression of IL-9R protein was obvious decreased in siIL-9R cells than sicontrol cells. (TIF 206 kb)
