# Supplementary figures and images for: Interleukin-9 promotes cell survival and drug resistance in diffuse large B-cell lymphoma
Source: J Exp Clin Cancer Res. 2016 Jul 1;35:106. doi: 10.1186/s13046-016-0374-3 (PMC4929715; doi:10.1186/s13046-016-0374-3)

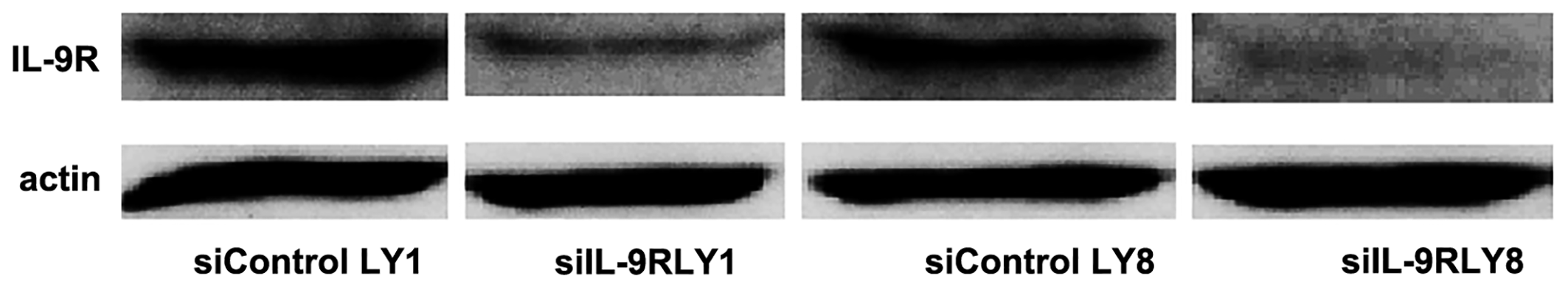

Supplement: Additional file 1: Figure S1. — IL-9R gene was knockout using lentivirus-mediated RNA interference. The efficiency of IL-9R knockdown was assessed by western blot analysis. The expression of IL-9R protein was obvious decreased in siIL-9R cells than sicontrol cells. (TIF 206 kb) [file 13046_2016_374_MOESM1_ESM.tif]
